# Hyperspectral Image Labeling and Classification Using an Ensemble Semi-Supervised Machine Learning Approach

**DOI:** 10.3390/s22041623

**Published:** 2022-02-18

**Authors:** Vidya Manian, Estefanía Alfaro-Mejía, Roger P. Tokars

**Affiliations:** 1 Department of Electrical and Computer Engineering, University of Puerto Rico, Mayaguez, PR 00681, USA; estefania.alfaro@upr.edu; 2 NASA Glenn Research Center, 21000 Brookpark Rd, Cleveland, OH 44135, USA; roger.p.tokars@nasa.gov

**Keywords:** hyperspectral images, semi-supervised learning, groundtruth, labeling, feature extraction, principal components analysis, normalization, image classification and reconstruction

## Abstract

Hyperspectral remote sensing has tremendous potential for monitoring land cover and water bodies from the rich spatial and spectral information contained in the images. It is a time and resource consuming task to obtain groundtruth data for these images by field sampling. A semi-supervised method for labeling and classification of hyperspectral images is presented. The unsupervised stage consists of image enhancement by feature extraction, followed by clustering for labeling and generating the groundtruth image. The supervised stage for classification consists of a preprocessing stage involving normalization, computation of principal components, and feature extraction. An ensemble of machine learning models takes the extracted features and groundtruth data from the unsupervised stage as input and a decision block then combines the output of the machines to label the image based on majority voting. The ensemble of machine learning methods includes support vector machines, gradient boosting, Gaussian classifier, and linear perceptron. Overall, the gradient boosting method gives the best performance for supervised classification of hyperspectral images. The presented ensemble method is useful for generating labeled data for hyperspectral images that do not have groundtruth information. It gives an overall accuracy of 93.74% for the Jasper hyperspectral image, 100% accuracy for the HSI2 Lake Erie images, and 99.92% for the classification of cyanobacteria or harmful algal blooms and surface scum. The method distinguishes well between blue green algae and surface scum. The full pipeline ensemble method for classifying Lake Erie images in a cloud server runs 24 times faster than a workstation.

## 1. Introduction

Hyperspectral imaging (HSI) provides a high density of spectral information in the hundreds of bands of the imaged material. Most modern hyperspectral sensors also have a high spatial resolution enabling the images to have a range of applications in agriculture, ecosystem monitoring, astronomy, molecular biology, biomedical imaging, geosciences, physics, and surveillance. Hyperspectral unmixing is the method of identifying the percentage of material or endmember contributions in each pixel, hence useful for material identification or detection. There are linear and nonlinear methods for hyperspectral unmixing [1]. They can be used to gain preliminary knowledge on the site before embarking on a field campaign. These images are particularly useful for informed decision-making on a terrestrial or aquatic ecosystem. 

Hyperspectral image classification requires preprocessing methods to reduce dimensionality and requires algorithms to solve the issues of few labeled samples, and low spatial resolution [2]. Traditionally, hyperspectral images have been classified using supervised, semi-supervised, and unsupervised Machine Learning (ML) methods. HSI classification is usually done after applying dimensionality reduction, feature extraction, and/or band subset selection. A review of the ranking, clustering, searching, sparsity, embedding, and hybrid scheme-based methods for band selection are given in [3]. A review of non-negative matrix factorization techniques and benchmark datasets for unmixing are presented in [4]. Low spatial rank tensor factorization methods are popular for unmixing hyperspectral images with mixed pixels [5]. ML approaches such as Random Forest (RF) and XGBoost have been applied in precision agriculture for the estimation of biomass [6]. HSI are also used for change detection in the ocean. A spatial–spectral attention network with PCA-based features is used for change detection [7].

The challenging problems with HSI classification are the unlabeled pixels and the high dimensionality of hyperspectral images. It is also expensive to assign labels to the pixels from field sampling requiring human supervision. To address the problem of unlabeled samples, ML algorithms have been developed which are described below. A graph-based semisupervised learning or ensemble label propagation method using spectral–spatial similarity measurements from a graph representation is proposed in [8]. Recently, Deep Learning (DL) methods are being developed and used for HSI classification [9]. Autoencoders have been used for hyperspectral unmixing and extended to the classification of HSI [10,11,12]. DL networks require a large number of labeled samples, which is overcome by few shot learning from spectral–spatial features, and training and testing using a 3D CNN in a metric space [13]. One of the disadvantages of using DL techniques is the computational complexity and cost. ML techniques are promising, but require pre-processing and feature extraction stages before training and validation. A local and global modeling approach for pseudo labeling using Active Learning (AL) is proposed in [14] for HSI classification. Tree-based approaches have gained attention in semi-supervised HSI classification. An ensemble semi-supervised random forest method is used for adaptively labeling unlabeled data and adding them to the training dataset [15]. AL and semi-supervised learning are combined to improve the performance of random forest method for HSI classification in [16]. The current ensemble classifiers and semi-supervised methods do not consider all the samples without labeling. The novelty of our ensemble semi-supervised scheme takes into account all the unlabeled samples in the HSI. Moreover, considering the computational complexity of DL networks, we propose a scheme for improving the performance of ML approach for HSI classification by image preprocessing using spectral textural and statistical feature extraction for image enhancement and semi-supervised ensemble labeling and classification in the following way:

- Unlabeled samples are labeled without a pre-trained labeled model by extracting spectral textural and statistical features and incorporating them in the image enhancement stage.
- The textural energy and statistical features computed in the image enhancement stage are input to a k-means clustering stage.
- The novel workflow consists of assigning labels to the unlabeled samples using spectral textural and statistical information in the unsupervised stage, followed by the application of an ensemble of four ML classifiers in the supervised stage, and a decision block that selects the best classifier for the classification of the image.

We apply our ensemble semi-supervised ML scheme for labeling and classification of hyperspectral images acquired over water bodies with Harmful Algal Blooms (HABs). HABs occur in fresh, marine (salt), and brackish (mixture of salt and fresh) water bodies around the world. They are caused by noxious and toxic phytoplankton, cyanobacteria, benthic algae, and microalgae. They are also produced by the overabundance of nutrients such as nitrates, ammonia, urea, and phosphates in the water. These nutrients runoff into the water from agriculture, fertilizers, and urban activity. The HABs lower oxygen levels in the water causing harm to organisms, animals, the environment, and the economy. The bloom lifespan lasts as long as there are favorable conditions but typically ranges from a few days to many months. HABs have been increasing in size and frequency worldwide, and it is caused by possible global climate change. Hence, HAB monitoring is key to the management of the health and utility of waterbodies. NOAA has used hyperspectral sensors to detect HABs in Lake Erie, one of the Great Lakes that border the U.S. and Canada. The hyperspectral camera collects information on the location, size, the concentration of the blooms, and types of algae [17]. The NASA Glenn Research Center (GRC) has developed an in-house hyperspectral camera, the airborne HSI2 that operates in the wavelength of 400 to 900 nm useful for HAB identification [18]. It can collect data at a high spatial resolution of 1 m, with the advantage of on-demand airborne flight paths not affected by cloud cover [19]. The HSI2 camera images have been used for assessing spatial and temporal variability of blue-green algae, chlorophyll, and temperature [20]. The airborne imagery serves as a complement to satellite-based measurements. HAB detection has been done using varimax-rotated principal components to isolate noise, extracting spectral components, and spatial patterns [21]. Satellite imagery from Sentinel-2A has been used for retrieval of chlorophyll-a concentration using empirical algorithms applied to the image bands, and an ensemble method. An ensemble is a set of base estimators that can be combined to make new predictions [22]. Moreover, Sentinel-2A images have been used for the estimation of chlorophyll-a concentration from regionally and locally adaptive models. Several empirical models were evaluated and found that the single global model constructed by the top-performing empirical algorithm performed best in estimating Chlorophyll-a concentration from both the multispectral and hyperspectral airborne images [23]. 

In this paper, we present a semi-supervised approach for labeling and classification of HSI that combines the best classifiers to provide optimal classification results. The rest of the paper is organized as follows. Section 2 presents an overview of the methodology and the algorithms used for preprocessing, feature extraction, clustering, and classification. It presents the ensemble ML models: (1) for labeling HSI in the absence of groundtruth data, which requires a preliminary clustering procedure, and (2) labeling and classification of HSI with groundtruth data. Section 3 presents results, while Section 4 discusses the results and compares them with those of state-of-the-art methods. The limitations and future work are presented here. The conclusions are provided in Section 5.

## 2. Materials and Methods

This section describes the images, and the methods used for preprocessing, labeling, and classification of the hyperspectral images. Two types of HSI are used: ones without groundtruth data and another with groundtruth data. The ones without groundtruth data are from airborne HSI sensors flown by NASA Glenn Research Center (GRC).

**Figure 1:**
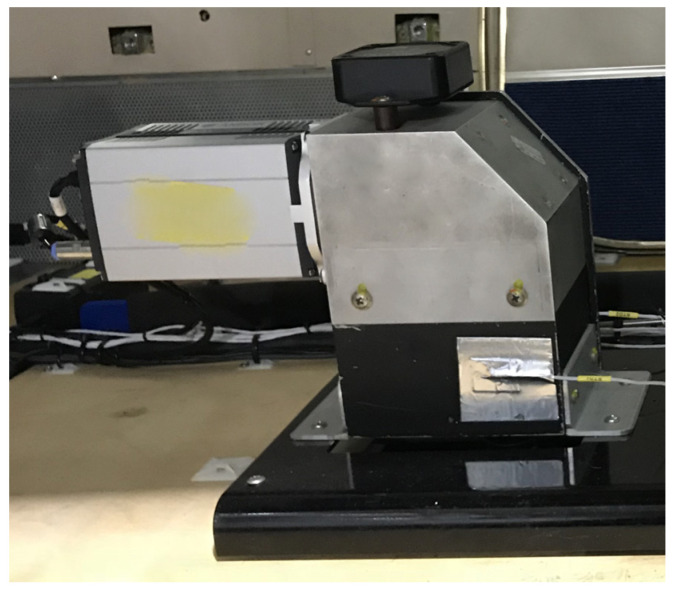
HSI2 installed on the NASA Twin Otter aircraft.

The processing of hyperspectral images involves calibration of the images in the laboratory and georeferencing of the data in flight. The calibration in the laboratory utilizes a known National Institute of Standards and Technology (NIST) calibrated radiance source to convert image intensity counts to radiance units. The calibration also utilizes a HgAr light source to convert the spatial pixel axis into known wavelength units. Additionally, in-flight O_2_ absorption lines fine-tune these wavelength calibrations to negate the effects of temperature and pressure differences. In-flight measurements of latitude, longitude, and attitude allow for georeferencing of the images. Figure 1 shows the HSI2 sensor installed on the NASA Twin Otter aircraft.

**Figure 2:**
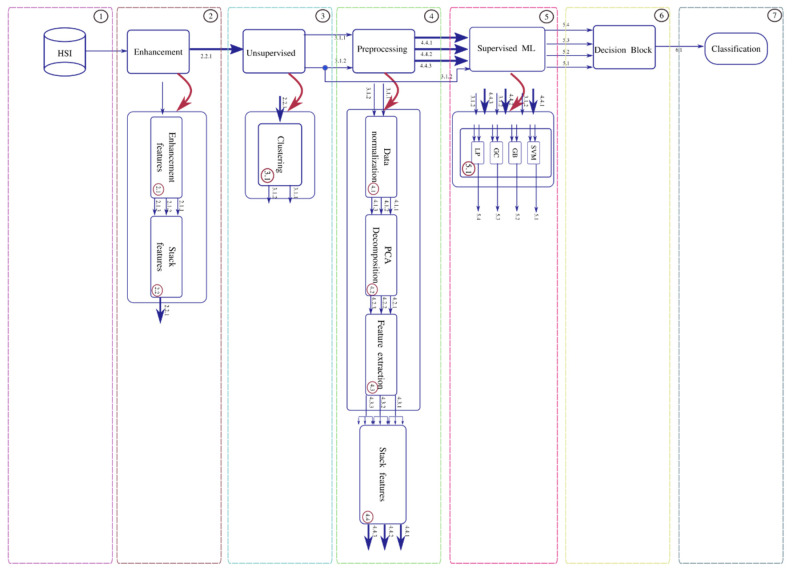
Block diagram for the semi-supervised hyperspectral image classification framework. The numbers on the arrows from top to bottom indicate the 3 types of scaling. The blue arrows represent the input and output for each stage, and the stages are represented by the blocks. The red arrow is a zoom into the corresponding stage showing what happens in that stage in detail.

We have used two HSI2 camera images from the Ohio Supercomputer Center (OSC) and one image developed by GRC for HAB monitoring in near real-time in Lake Erie [19]. The two HSI2 images are one-meter resolution with 51 bands from 400 to 900 nanometers of size 5000 rows and 495 columns, and the CyanoHAB hyperspectral image has 170 bands from 400 to 900 nm with a spectral resolution of 2.5 nm, also with a spatial resolution of 1 m. Figure 2 shows the block diagram for the proposed semi-supervised classification scheme.

The proposed semi-supervised HSI classification workflow is illustrated in Figure 2. The workflow has seven stages. The HSI2 images have NaN entries for some pixel data points. The sunlight reflects off the water causing imager saturation by glare or speckle. Hence, NaN is inserted at these data locations. The first stage corresponds to the input and is the hyperspectral image, the image is read and is processed using a data frame structure and the NaN values are replaced by the mean of the five neighborhood pixels. After this filtering process, the enhancement stage has two sub-processes 2.1 and 2.2 (shown in Figure 2). In Section 2.1 relevant features are extracted using the stacked 51 bands of the image using the first-two statistical moments (mean, standard deviation) and texture information as an energy feature. These features are described below:

(1) $$\xi ={\sum_{n=1}^{N_{1}N_{2}}\left|x[n]\right|}^{2}$$

is the energy. *N*_1_ and *N*_2_ are the batch size [24]. The mean is computed as:

(2) $$\mu =\frac{1}{N_{1}N_{2}}\sum_{j=1}^{N_{2}}\sum_{i=1}^{N_{1}}x\left[i\right]\left[j\right]$$

and the standard deviation feature is computed as:

(3) $$\sigma =\sqrt{\frac{1}{N_{1}N_{2}}\sum_{j=1}^{N_{2}}\sum_{i=1}^{N_{1}}{\left(x\left[i\right]\left[j\right]-\mu \right)}^{2}}$$

In the 2.2 sub-process, the enhancement vectors are stacked. The 3 arrows indicate the 3 features which are then stacked into one data frame. In the 2.2 sub-process, the stacked vectors are then input to the unsupervised stage. In the 3.1 sub-process, the label assignment is made. The data preparation for this stage requires a 1-D tensor representation of the image. The experiment consists of various trials of cluster numbers, k = 2 to 5, to result in an output image label representation from the original image after the enhancement stage. Once the best label assignment for the Lake Erie image is determined, we have the data and the corresponding labels. Since the images do not have specific groundtruth data, the unsupervised stage produces a label representation of the original image for the best number of clusters. The next is stage 4 processing which includes 4 sub-processes. Sub-process 4.1 is a data normalization process using three different kinds of normalization: normalization scaling (ns), maximum scaling (ms), and scaling (sc). After the data normalization process, sub-process 4.2 is PCA decomposition and selection of 3, 5, or 7 bands. In sub-process 4.3, the feature vectors **ft** from the enhancement stage are computed. In sub-process 4.4, the resulting vectors are stacked into an array **Y** and are concatenated with the labels provided by the unsupervised stage. Stacked vectors and the labels are then input to the process 5, supervised Machine Learning (ML) stage.

Stacked vectors **Y** and the labels go through a batch selection process before being input to the supervised ML stage. The Supervised ML stage has four machine learning techniques in an ensemble configuration: Support Vector Machines (SVM), Gradient Boost Classifier (GB), Gaussian Classifier, and a Linear Perceptron (LP) [25]. SVMs represent the training samples as points in p-dimensional space, mapped so that the samples of the data classes are separated by a (p-1) dimensional hyperplane. The hyperplane is chosen such that it maximizes the margin on either side of the hyperplane between two classes. Hence, SVM performs binary classification but can be extended to multi-class problems. Gradient boost classifiers combine many weak learning models to create a strong predictive model. It minimizes a loss function by iteratively choosing a function that points towards the negative gradient. A Gaussian classifier is a naïve Bayes classifier. It is a generative approach that models the class posterior and input-class conditional distribution. The LP is a linear feedforward network with an input and an output layer. 

The stage 6 process is the decision block that decides the best classifier based on the classification accuracy results obtained from testing the trained models. The final classification stage 7 receives the decision block results and labels the HSI pixels to fixed class labels. The classification stage results are also evaluated using three metrics. They are the classification accuracy, F1-score, and the Structural Similarity Index Metric (SSIM). The SSIM compares the reconstructed image with the labeled image and rates how good the reconstructed image from the classification is compared to groundtruth labeled image. The SSIM is given by:

(4) $$\mathrm{SSIM}(x,y)=\frac{\left(2\mu_{x}\mu_{y}+C_{1}\right)\left(2\sigma_{xy}+C_{2}\right)}{\left(\mu_{x}^{2}+\mu_{y}^{2}+C_{1}\right)\left(\sigma_{x}^{2}+\sigma_{y}^{2}+C_{2}\right)}$$

where *x* and *y* are two non-negative image signals, *µ_x_* and *µ_y_* are their means, and *σ_x_* and *σ_y_* are their standard deviations, *σ_xy_* the correlation, and *C*_1_ and *C*_2_ are constants [26,27]. The SSIM is widely used for the assessment of image quality and it satisfies the conditions of symmetry, boundedness, and unique maximum.

We used Amazon Web Services (AWS) [28] to run the training models written in Python for classification of the two HSI2 Lake Erie images. AWS hardware resource is an EC2 instance of type R5 extra large which has six Virtual CPUs (VCPUs), and 32 GB RAM. This particular instance provides optimized memory computing.

### 2.1. Workflow for Supervised Classification of Jasper Image

**Figure 3:**
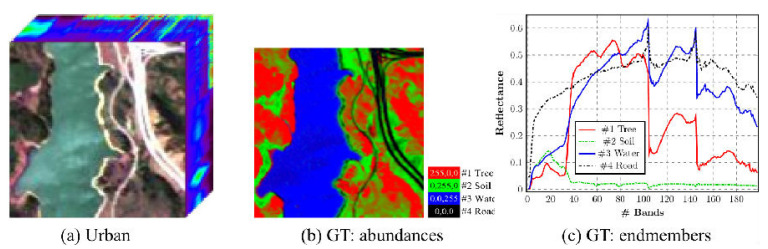
Jasper HSI (**a**) original image, (**b**) Groundtruth abundances, (**c**) Groundtruth endmembers.

**Figure 4:**
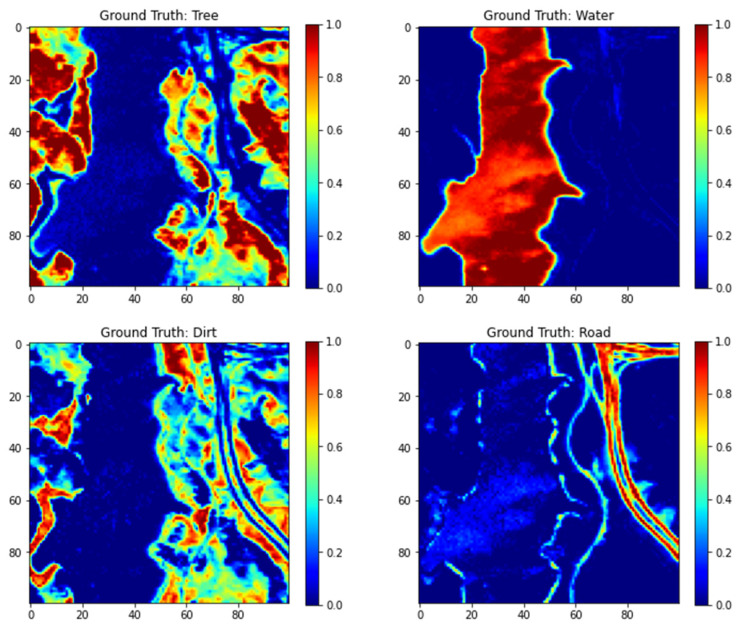
Endmember abundances for the four endmembers for Jasper image.

We have used the Jasper HSI, because it is similar to the Lake Erie image as it has land cover and an inland water body. Figure 3 shows the Jasper image along with the groundtruth. The Jasper image has 100 rows, 100 columns, and has 224 bands. Figure 4 shows the four endmember abundances for the materials present in the Jasper image. The endmembers are road, soil, water, and tree. We did not consider the road class because of an insufficient number of pixels for training. The available groundtruth has endmember abundances for each of the pixels. In [29] random labeling of the HSI pixels is used for creating labels. Here, we conduct two classification experiments by generating labels based on groundtruth endmember abundances. For the first experiment, to perform a fixed classification of each pixel to a particular class, we created three labels for each pixel from the endmember abundances as strongly belong, weakly belong, and does not belong to one of the three original groundtruth classes. If the fractional abundance is greater than 0.8, then the pixel is labeled as strongly belonging to the class. If the fractional abundance is less than 0.8, then the pixel weakly belongs to the class, and if the abundance is 0 the pixel does not belong to the class. All the four machines are trained with training batches for the three groundtruth classes and for the three labels for each of the three groundtruth classes resulting in training of nine classes. We also conduct a second classification experiment with two labels for pixels. The pixel is labeled as strongly belonging to the class if the abundance is less than the groundtruth maximum value for the class and greater than 0.4. If the pixel value is greater than the minimum groundtruth value and less than 0.4, it is labeled as not belonging to the class, resulting in the training of 6 classes. For both the experiments, 10 fold cross-validation is done which results in the training of a total of 90, and 60 models for both experiments, respectively. We have effectively converted an unmixing problem into a classification problem by assigning fixed labels to pixels with fractional abundances by thresholding. The procedure for preprocessing and extraction of batch sizes for training and testing are explained below.

**Figure 5:**
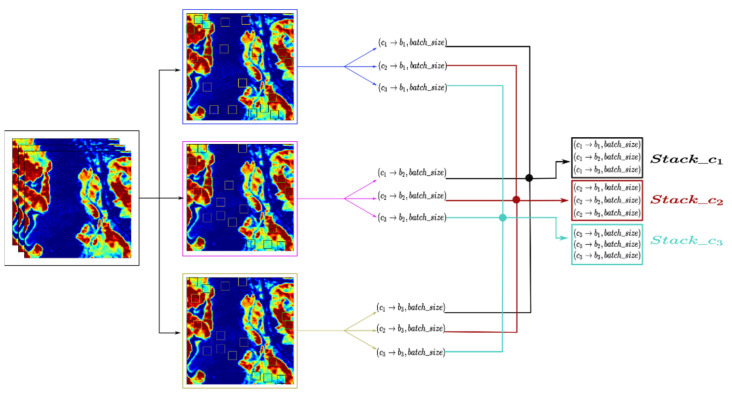
Batch size selection process for the three PCA bands from the Jasper HSI.

Firstly, PCA is applied to the Jasper image, and three, five, and seven dominant PCA bands are selected. The batch selection process consists of the random extraction of parts of the image by class. The batches are divided into groups for training, testing, and left-over data. Batch sizes for the training data are 820, 1000, and 1500 pixels. The data is split into training data, testing data corresponding to the same selected batch size as training data, and the remaining data not used for training or testing is used only for the image reconstruction. This data is around 400 pixels. The training is done with less than 2% of the pixels of Jasper HSI for each class. Figure 5 explains the batch size extraction process for three PCA bands with min–max scaling. The experiment is repeated with max-scaling and normalization. Finally, the batches are stacked for training the models. For two labels (strongly belong, and does not belong), the training batch sizes are (6 × 820 pixels), where 6 corresponds to two batches for each of the three PCA bands. The six batches per class are stacked together for training the models for all three classes. The testing batch sizes are (9 × 820 pixels) where 9 corresponds to three batches for each of the three PCA bands which are stacked together for testing for the three classes. There is a remaining 980 pixels of left-over data which is used for image reconstruction. The experiment is repeated for batch sizes of 1000 and 1500 pixels. For three labels (strongly belong, weakly belong, and does not belong), the batch sizes are smaller: 300, 500, and 600 pixels. The training is done on the features extracted from the batches.

**Figure 6:**
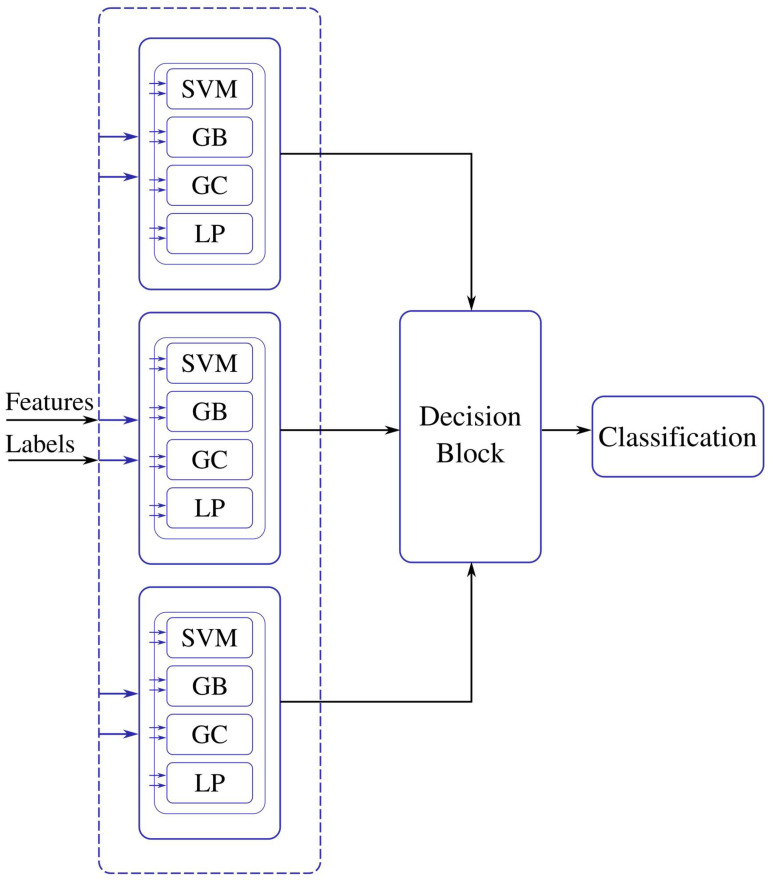
Energy, mean, and variance features are calculated from the Jasper HSI training samples and are input to the ML algorithms. The decision block selects the best machine for each class and uses the selected machines to label the testing samples.

The features are energy, mean, and standard deviation which are calculated on the batches of pixels. The ML models are trained with the computed features. The ensemble model for the training process for the three classes, trees, water, and soil, is shown in Figure 6. The labeled testing pixels are then used to reconstruct the classified Jasper image with color code for each class. 

Pseudocode description of the algorithms for the image enhancement features block, supervised ML block, and decision block are given below.

**A.**  **Pseudo code feature enhancement block**

**Input:** Hyperspectral Image

**Output:** Stacked vector Enhancement

**Begin:**

   compute the energy feature using Equation (1)

   compute the mean using Equation (2)

   compute the standard deviation using Equation (3)

   concatenate the energy, mean and standard deviation in to a data frame

**Return** Stacked enhancement vector

The enhancement features block is applied to obtain the spectral features representation. The input is the 1-D reshaped hyperspectral image vector placed as columns for each of the bands, then the energy, mean, and standard deviation feature are extracted. Finally, the data is stacked in to a data frame.

**B.**  **Pseudo code supervised machine learning block**

**Input:** dataset train (data), label for dataset train (label), tolerance, kernel, depth, estimators

**Output:** Models

**Begin:** Initialize variables for accuracy, F1 score, confusion matrix for the models (metrics)

**For** 10-fold cross validation of the data

   compute **SVM** Model using data, label, and tolerance

   compute **GB** Model using data, label, estimators, and depth

   compute **LP** Model using data, label, and tolerance

   compute **GC** Model using data, label, and kernel

   compute accuracy score for the four models

   compute F1 score for the four models

   compute confusion matrix score for the four models

   save (SVM Model, GB Model, LP Model, GC Model) 

   append accuracy, F1-score, confusion matrix

**Return** Models, metrics

The unsupervised machine learning block proposed is composed of four machine learning methods: SVM, GB, GC, LP. The models are trained using a 10-fold cross-validation methodology. Then, the input of the machine learning blocks is the selected training data, the respective labels, and the tuning parameters. The tuning parameters are configured for each machine learning technique as follow:

SVM is set using a linear kernel, and hinge as a loss function and tolerance values of 1 × 10^−3^. The Gradient Boosting parameter is the depth of the individual regression estimator which is set to 10, the number of boosting stages is 100, and the learning rate for each tree is 1.0. The LP classifier is set to tolerance or stopping criteria of 1 × 10^−5^. The Gaussian classifier is set with the RBF kernel using L-BFGS quasi-Newton methods as an optimization function.

**C.**  **Pseudo code decision block**

**Input:** data_test (batch_size, features), label (batch_size), models

**Output:** Best classifiers

**Begin:** Initialize dictionary metrics variable (accuracy, F1 score, confusion matrix, training data, predicted labels), maximum accuracy variable

**For each** folderModels

   **For each** Model

      load model

      compute accuracy 

      compute F1 score

      compute confusion matrix

      append accuracy, F1 score, confusion matrix, model, and variables in dictionary metrics

   concatenate dictionary metrics in a pandas data frame

obtain the best model classifier using the accuracy criteria

**Return** best classifier

The above pseudocode procedure is for the principal blocks of the workflow in Figure 2 and Figure 6. The rest of the blocks that include preprocessing methods for scaling, and dimensionality reduction using PCA are straightforward to compute.

## 3. Results 

This section presents and discusses the results of applying the ensemble method for the labeling, classification, and reconstruction of the HSI2 images and Jasper hyperspectral images.

### 3.1. Classification and Reconstruction of HSI2 Images

**Figure 7:**
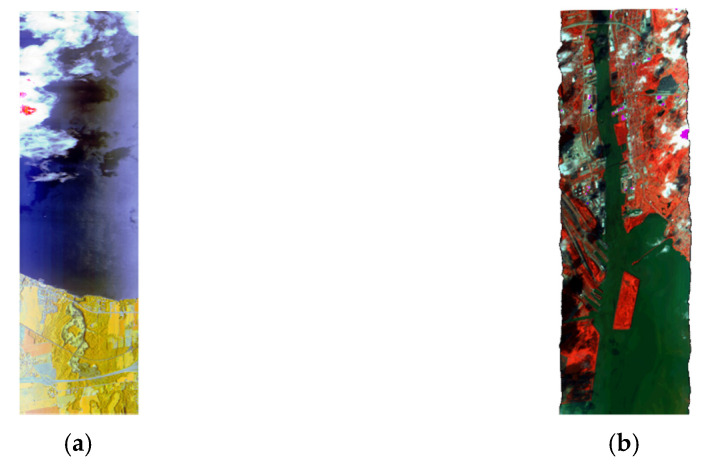
HSI2 Hyperspectral images over Lake Erie (**a**) Image 1 (white—clouds, blue—water, yellow—land), and (**b**) Image 2 (white—clouds, green—water, red—land).

The semi-supervised classification pipeline is applied to two HSI2 images over Lake Erie. The HSI2 images (Image 1 and Image 2) are shown in Figure 7. Image 1 is of size 3270 × 960, where 3270 is the number of lines, and 960 is the number of samples per line. Image 2 in Figure 7b is of size 4444 × 960, where 4444 is the number of lines, and 960 is the number of samples per line. The semi-supervised classification scheme shown in Figure 2 is applied to the images shown in Figure 7a,b.

**Figure 8:**
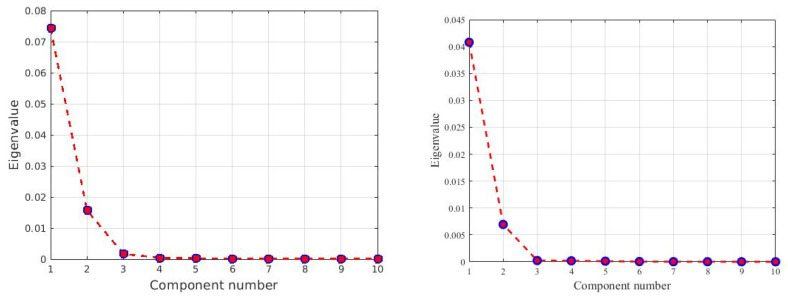
Scree plot of the contribution of each principal component bands for the two hyperspectral images in Figure 7 (Left Image 1 and Right Image 2).

The unsupervised stage for segmenting the image into clusters is applied for a choice of 2, 3, 4, and 5 clusters. This stage performs k-means clustering after image enhancement using the standard deviation and energy features. This combination and 3 numbers of clusters give the best results for labeling and obtaining the groundtruth image. Following unsupervised classification which identifies the best number of clusters image preprocessing is performed. PCA is used for selecting the best number of a subset of bands. There are 51 bands in the HSI2. The covariance matrix of the image and its Eigenvalues are computed. Figure 8 shows the percentage of contribution of the first ten bands to the Eigenvalues of the covariance matrix of the image. It can be seen that all the energy is compacted in the first three bands of the image. 

Following preprocessing, extraction of the three mean, standard deviation, and energy features is performed. For supervised classification, all the three features are used. The features are stacked for training the four ML methods. The Ensemble of the four machines is applied to HSI2 images 1 and 2 in Figure 7. The decision block loads all the models of the 10 fold cross-validation process, and classifies the images with all the models, and choose the best model using the classification accuracy as the selection metric. 

Table 1 and Table 2 shows the accuracy and F1 score obtained from 10-fold cross validation for three clusters with 3, 5, and 7 PCA bands, using the three scaling methods of standardization normalization (ns), max scaling (ms), and min–max scaling(sc) for the HSI2 Image 1 in Figure 7a, and HSI2 Image 2 in Figure 7b, respectively. The three clusters are land, water, and clouds. Table 3 and Table 4 show the accuracy and F1 score obtained from 10-fold cross validation for three clusters with 3, 5, and 7 PCA bands, using the three scaling methods of normalization scaling, min–max scaling, and max scaling for the HSI2 Image 2 in Figure 7b. 

**Figure 9:**
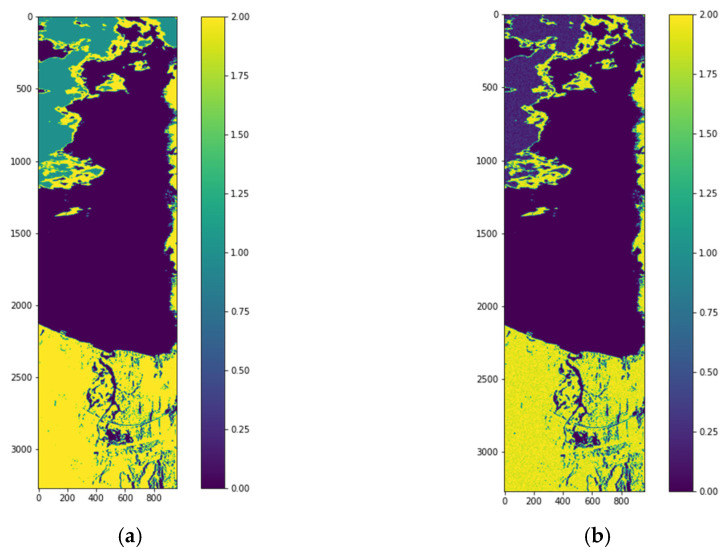
(**a**) HSI2 Image 1 with labels for 3 classes using the unsupervised stage (k-means clustering method), (**b**) Reconstructed image from classified samples using the supervised stage.

The models are trained with the extracted features for a batch size of 1500 for HSI2 image 1 and image 2 for three classes. For both images, the best batch size is found to be 1500 pixels compared to 1000 pixels batch size. The trained models are then used to classify the images into three classes. The classified images are reconstructed. The best accuracy is obtained with the GB model and 3 PCA bands for both images. The reconstructed labeled image and reconstructed classified image for HSI2 image 1 are shown in Figure 9a,b, respectively. The SSIM between the labeled image and the reconstructed image is 0.6743.

**Figure 10:**
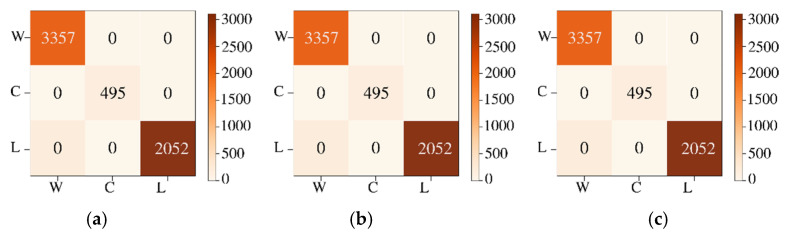
Confusion matrices for classification of HSI2 Image 1 into 3 classes for a batch size of 1500 using 3 PCA bands with the three types of scaling methods (**a**) normalization scaling (ns), (**b**) maximum scaling (ms), (**c**) scaling (sc). The three classes are indicated as W—water, C—clouds, L—land.

Figure 10 shows the confusion matrices for the reconstruction of HSI2 Image 1 using 3 PCA bands with the three types of scaling methods. All of the scaling methods give 100% accuracy using GB classifier, and second best classifier is GC.

**Figure 11:**
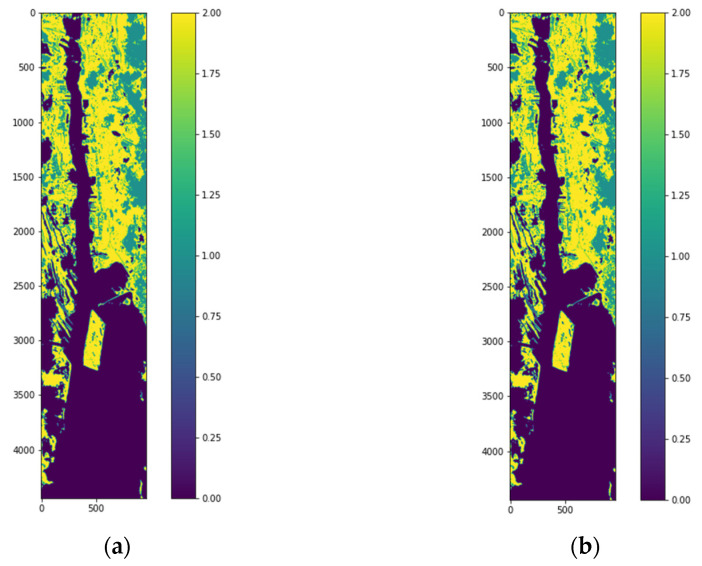
(**a**) HIS2 Image 2 with labels for 3 classes using the unsupervised stage (k-means clustering method), (**b**) Reconstructed image from classified samples using the supervised stage.

**Figure 12:**
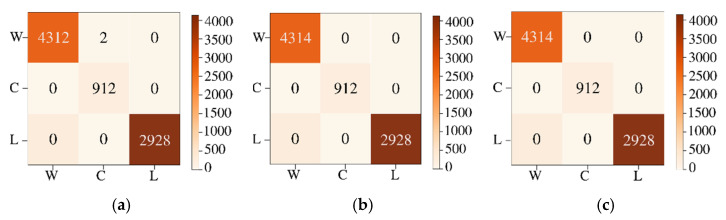
Confusion matrices for classification of HSI2 Image 2 into 3 classes for a batch size of 1500 using 3 PCA bands with the three types of scaling methods (**a**) normalization scaling (ns), (**b**) maximum scaling (ms), (**c**) scaling (sc).

The labeled image and classified reconstructed image for HSI2 image 2 are shown in Figure 11a,b, respectively. The classified image of HSI2 image 2 using 3 PCA bands and maximum scaling (ms) gives the best similarity with the labeled image with the SSIM being 1.0. Figure 12 shows the confusion matrices for the reconstruction of HSI2 Image 2 using 3 PCA bands with the three types of scaling methods. Overall, the scaling and maximum scaling methods give 100% accuracy, and the normalization scaling gives 99.95% accuracy. For HSI image 1, the highest accuracies are obtained for using 3 PCA bands and maximum scaling, and for HSI image 2, the highest accuracies are obtained for using 3 PCA bands and normalization scaling. The confusion matrices in Figure 10 and Figure 12 have different number of testing samples, as the HSI2 images 1 and 2 are of different size.

### 3.2. Classification of Cyanohab from Lake Erie Image

**Figure 13:**
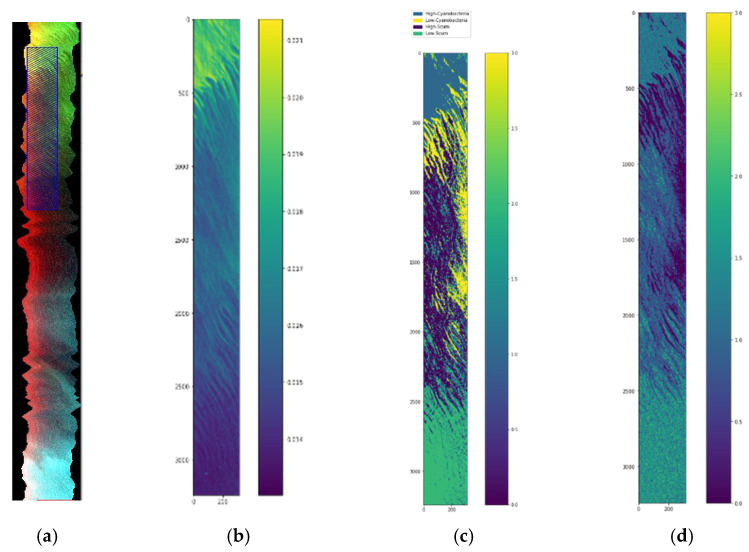
(**a**) Hyperspectral image of Lake Erie with extracted ROI shown as blue rectangle, (**b**) Zoomed ROI subimage, (**c**) Output image from unsupervised stage with four clusters, (**d**) Classified reconstructed image using 3 PCA bands and normalization scaling (Legends are the same as image in (**c**)).

We use another HSI image shown in Figure 13 to show that the ensemble semi-supervised scheme can identify blue green algae or cyanoHAB (cyanobacteria) from other materials in the lake. This image was acquired using a different sensor than HSI2, the data has a different format. The image is of size 5000 lines, with 496 samples per line. We used ENVI to obtain the ROIs for the cyanobacteria and surface scum. This image shows higher concentrations of cyanobacteria and also surface scum. A Region of Interest (ROI) with a high concentration of cyanobacteria in the East side of the lake, highlighted by a blue rectangle in Figure 13a is extracted. The ROI image is of size 3240 lines, with 311 samples per line. The ROI image is shown enlarged in Figure 13b. The image is stored in ‘tif’ format in 170 bands and the proposed workflow shown in Figure 2 is applied, similar to the classification of HSI2 images. The enhancement stage performs feature extraction of the textural energy and statistical mean and standard deviation features. Then, the vectors are stacked using a Pandas data frame structure. The next stage is the unsupervised stage for label assignment in the image using a k-means clustering that takes as input the stacked features vectors. The output of this block are the labels and data. The labeled image with four clusters is shown in Figure 13c. A preprocessing stage is performed using data normalization followed by feature extraction. The previous outputs are the inputs for the supervised machine learning ensemble trained with a batch size of 1000 by 3 features similar to the previous experiment on Lake Erie HSI2 images. After training, the decision block decides the best of the four machines using majority voting, using which the final classification and reconstructed image are obtained. The classified reconstructed image is shown in Figure 13d.

The classification accuracies using the supervised stage of the ensemble method for the 3 scaling methods, and 3, 5, and 7 PCA bands are given in Table 5. As can be seen, the three PCA bands result in higher accuracies using the Gradient Boosting classifier. The F1 shores are given in Table 6. 

**Figure 14:**
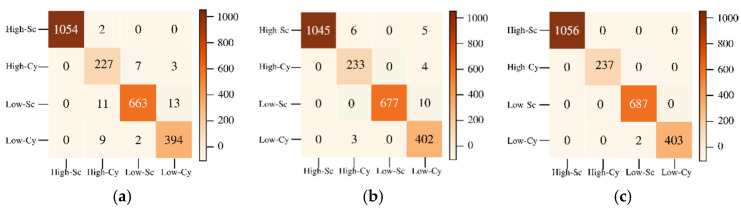
The confusion matrices for 4 labels High Scum, High Cynanobacteria, Low Scum, and Low Cyanobacteria pixels of the CyanoHAB image for three types of scaling- (**a**) normalization scaling (ns), (**b**) maximum scaling (ms), and (**c**) scaling (sc).

The confusion matrices for the 4 classes are given in Figure 14. The classified reconstructed image shown in Figure 13d has the same color legends as Figure 13c.

### 3.3. Classification and Reconstruction of Jasper Image

**Figure 15:**
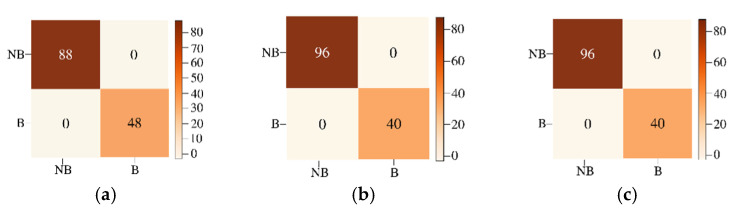

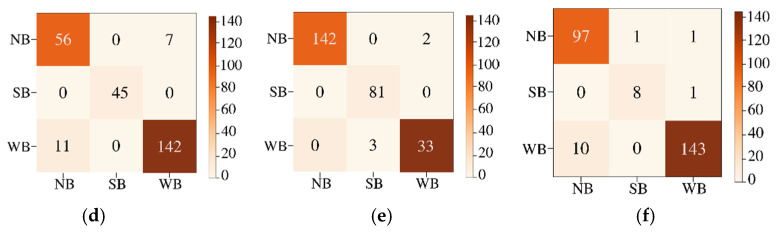
The confusion matrices for 2 labels Not Belong (NB) and Belong (B) given to the (**a**) trees, (**b**) water, and (**c**) soil pixels of the Jasper HSI in the top row. Bottom row shows the confusion matrices for 3 labels Strong Belong (SB), Weak Belong (WB), and Not Belong (NB) given to the (**d**) trees, (**e**) water and (**f**) soil pixels of the Jasper HSI.

Jasper HSI has 4 different materials with mixing in each pixel. We did not consider the road class because of insufficient data for training. The three considered classes are trees, water, and soil. The Jasper image pixels have been classified into subcategories: Belong (B) and Not Belong (NB) and to three subcategories: Strong Belong (SB), Weak Belong (WB), and Not Belong (NB) to give fixed labels to the groundtruth pixels with fractional abundances. For two labels within the three classes of trees, water, and soil, 2 × 2 confusion matrices are obtained for each of the three classes, and for three labels within the three classes, 3 × 3 confusion matrices are obtained (shown in Figure 15) for each of the three classes. For the two subcategories experiment, we have a total of 136 testing samples, and for the three subcategories experiment, we have a total of 261 testing samples.

**Figure 16:**
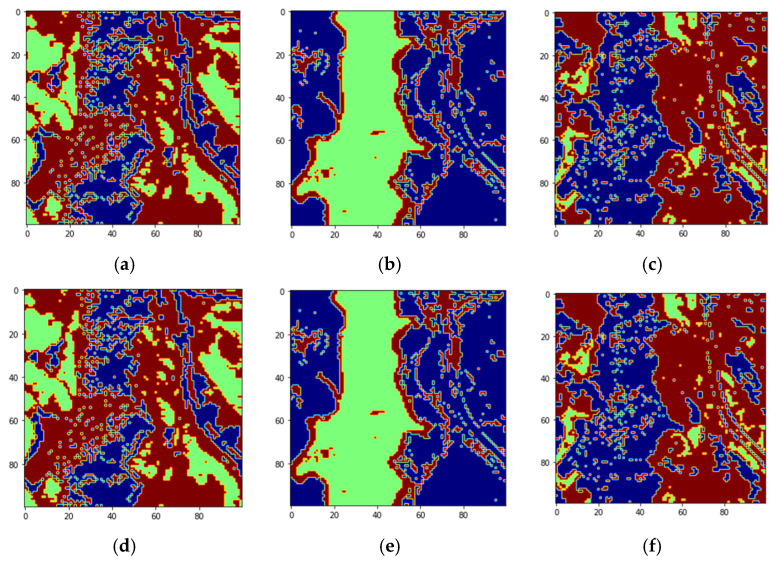
(**a**) Tree class reconstructed image with label 0 (blue) for pixels Not Belonging to tree class, label 1(green) for Strong Belonging pixels to tree class, and label 2 (brown) for pixels Weakly Belonging to tree class. (**b**) Water class reconstructed image with label 3 (blue) for pixels Not Belonging to water class, label 4 (green) for Strong Belonging to water class, and label 5 (brown) for pixels Weakly Belonging to water class, (**c**) Soil class reconstructed image with label 6 (blue) for pixels Not Belonging to soil class, label 7 (green) for pixels Strongly Belonging to soil class, and label 8 (brown) for pixels Weakly Belonging to Soil class, (**d**) Original groundtruth for tree class, (**e**) Original groundtruth for water class, and (**f**) Original groundtruth image for soil class.

We divided the data into batches of training and testing sizes and computed the classification accuracies using 10-fold cross validation. Figure 16 shows the classified images and the original groundtruth endmembers for each of the classes for classifying with three labels per class.

Table 7 and Table 8 show the accuracy and F1 score obtained from 10-fold cross validation for three clusters with 3, 5, and 7 PCA bands, using the three scaling methods of standardization normalization, min–max scaling, and maximum scaling for the Jasper HSI. The structural similarity index measure (SSIM) between the original and reconstructed image pixels is 1.0 for the tree, water, and soil classes. Best results are obtained with three PCA bands and maximum scaling. The batch sizes for training and classification for three labels per class are 300, 500, and 600. The best batch size is found to be 300 pixels.

**Figure 17:**
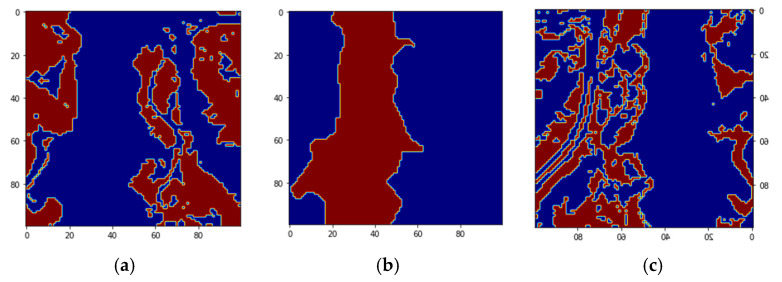

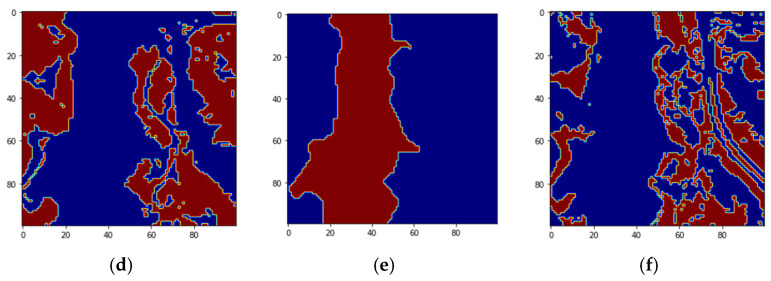
(**a**) Tree class reconstructed image with label 0 (blue) for pixels Not Belonging to tree class, label 1(brown) for pixels Belonging to tree class. (**b**) Water class reconstructed image with label 3 (blue) for pixels Not Belonging to water class, and label 4 (brown) for pixels Belonging to water class, (**c**) Soil class reconstructed image with label 6 (blue) for pixels Not Belonging to soil class, and label 7 (brown) for pixels Belonging to soil class, (**d**) Original groundtruth for tree class, (**e**) Original groundtruth for water class, and (**f**) Original groundtruth image for soil class.

The Jasper image is also classified into two labels per pixel versus Not Belong and Strong Belong by thresholding the fractional abundances as discussed in Section 2.1. The batch sizes for training and classification for two labels per class are 820, 1000, and 1500. The results of the reconstructed images for each endmember are shown in Figure 17. The SSIM for these reconstructions is also 1.0 giving the highest similarity between original and reconstructed images. 

## 4. Discussion

### 4.1. Discussion of Ensemble Model Results for HSI2 Images of Lake Erie

The semi-supervised ensemble method pipeline is larger for Lake Erie images because we do not have the labeled groundtruth data. The labeled data has to be created using the unsupervised stage of the pipeline. Moreover, the image enhancement stage makes use of all the 51 bands of the original image to compute the features that are input to the unsupervised stage. The enhancement stage is important as it improves the labeling of the original HSI dataset. Both images are labeled by the unsupervised stage into 3 classes: clouds, land, and water. The supervised stage implements four ML models and the output classified images are obtained after 10-fold cross validation. The best batch size is 1500 pixels stacked for the 3 features. The SSIM is 0.6743 for Lake Erie Image 1 while it is 1.0 for image 2 which has more land cover than image 1. This is because of the higher cloud cover in image 1. Optical remote sensing imagery has the problem of cloud cover and thresholding methods are applied for their removal from hyperspectral imagery [30]. Onboard spectral–spatial method is proposed in [31] for cloud detection. A deep learning neural network method is proposed for cloud detection in [32]. Our method can be used for masking and filtering cloud cover pixels before classification of the image. The advantage of our ensemble method is that the identification of cloud pixels is part of the labeling process in the pipeline, which is followed by supervised classification using the ensemble ML technique. 

### 4.2. Discussion of Ensemble Model Results for CyanoHAB Image of Lake Erie

**Figure 18:**
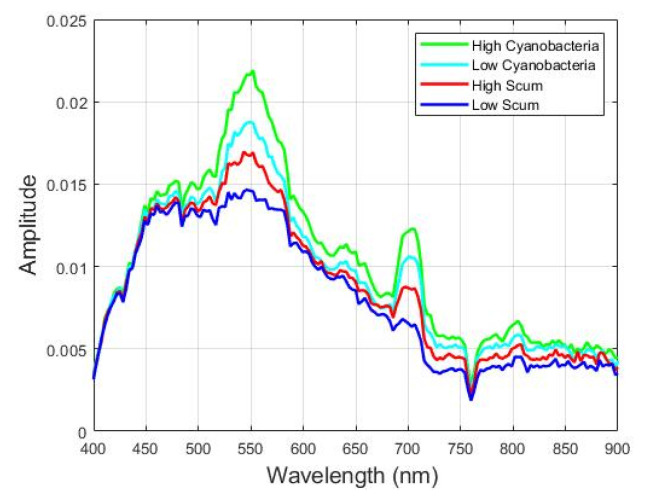
Spectral signature profiles of the CyanoHAB found in the Lake Erie Hyperspectral image.

The spectral signature of pixels in the four clusters from the ROI image in Figure 13c is shown in Figure 18. The bands 679 nm, 664 nm, and 709 nm, and the bands 667 nm and 858 nm are used to calculate the Cyanobacteria Index (CI) and Surface Scum Index (SSI), respectively in [19]. As can be seen from the output of the unsupervised stage the regions of High CyanoHAB, Low CyanoHAB, High Scum, and Low Scum are identified correctly compared to the images obtained from the CI and SSI in [19]. The classification accuracies for the supervised classification of CyanoHAB is 99.92%. Our classification of High CyanoHAB, High Scum, and Low Scum are good, but the accuracy is low for low cyanobacteria concentration. The classification of the low cyanobacteria class can be improved by spectral feature extraction. Our semi-supervised ensemble scheme can be used for the identification of cyanobacteria from hyperspectral images in an automatic manner without human intervention and the need for labeled samples. Moreover, the CI and SSI give a fractional index of the materials with one image per material. While our classification pipeline gives fixed labels for each pixel which will be more useful for water management as they know definitely which areas pertain to harmful cyanobacteria, and which are safer for recreation and other activities.

### 4.3. Discussion of Ensemble Model Results for Jasper HSI

For two subclasses by label, the classification results were compared for using three, five, and seven PCA bands. The best classification performance was obtained with the GB classifier, with three PCA bands giving an accuracy of 91.57%, and 100%, 100% for the tree, water, and soil classes, respectively. For the tree and soil classes a better accuracy is obtained with five PCA bands. On the other hand, for water classification, the performance is better with seven PCA bands. For larger batch sizes, e.g., 1000 and 1500, lower number of PCA bands such as three, gives an accuracy of 100% for the three classes with GB classifier. For the three labels per class configuration of SB, WB, and NB, three PCA bands for a batch size of 300 gives the best classification accuracy of 95.02%, 96.23%, and 89.27% for the tree, water, and soil classes, respectively, using the GB classifier. For the water class, we obtained better accuracy of 99.07% using a batch size of 600. The three labels per class configuration also gives a SSIM of 1.0 for the reconstructed image compared to the original groundtruth image. The best results for the Jasper image can be summarized as the use of three PCA bands with min–max scaling and GB classifier. The GB is found to be the best classifier as it is based on decision trees and it combines many weak learners to create a strong predictive model.

Jasper dataset has four endmembers’ contributions corresponding to tree, soil, water, and road. A graph-based architecture is proposed in [33] to classify the endmember contributions and, the authors compare the classification performance with ML techniques such as SVM, KNN, LDAKNN, PCAKNN, KPCAKNN, LDASVM, PCASVM KPCASVM, Convolution Neural Network (CNN), the abbreviation Linear Discriminant Analysis (LDA), Kernel PCA (KPCA) in the machine learning methods means the preprocessing step before the classification techniques. The accuracy measurements for Jasper image for classification into the endmembers contribution by class is as follows: Soil Class obtained 100% accuracy results using the PCAKNN method and SVM 99.905%, for water, and TLM-2 classifier obtained 98.959%, Finally, for tree class, TLM-2 obtained 97.622%. From our experiments, in the separate analysis for three classes using a labeled subset of non-belong (NB), strong-belong (SB), and weak-belong (WB), and using the feature extracted from three PCA bands, we obtained the following accuracies for three classes: trees 95.02%, water class 96.93%, and soil class 89.27% for the GB classifier. On the other hand, for two classes using as a label sub-set of non-belong (NB) and belong (B), we obtained 91.67%, 100.00%, and 100.00% for the three classes of trees, water, and soil, respectively. We improve the results compared to the method proposed in [33] for our two sub-labels approach. The best scaling method for Jasper dataset was the min–max-scaling. Our ensemble method improves the water and soil classification accuracies using 24.6% of the dataset for training and the remaining data for testing. In [29], the authors propose a Kernelized Extreme Learning Machine (K-ELM) using 2000 samples for training and the obtained accuracy score for a groundtruth labeling in the re-testing procedure for road, soil, water, and trees as: 84.7%, 98.06%, 69.4%, and 71.1% by class which are lower than the accuracies obtained by our ensemble method, and also requires a larger number of training samples.

The ensemble model can handle unlabeled samples. However, it needs sufficient unlabeled samples for training the machines. Since there are four machines involved the model is time-consuming. In the cloud server, the model takes 6 h 47 min for classifying the Lake Erie images which are still faster than a DELL desktop computer which takes about a week to classify one image. Currently, the model classifies pixels as belonging to particular classes, the future work will involve developing the model to determine fractional abundances of each pixel. Moreover, the future work will involve optimizing the training to work with fewer unlabeled samples using other machines such as DL networks.

## 5. Conclusions

A semi-supervised ensemble method is presented for labeling pixels in an HSI and classifying the image. The method performs well for airborne HSI over Lake Erie and the Jasper benchmark HSI. In the absence of groundtruth, this method can be used as a preprocessing step for labeling pixels and creating groundtruth data. Moreover, the unsupervised stage effectively detects cloud pixels in the HSI and can be used for cloud removal. The method is able to identify cyanobacteria and other water pollutants from HSI. As with any ML method, sufficient training samples are necessary for adequate training of the machines. The best normalization scheme is found to be maximum scaling, and the number of PCA bands depends on the spectral bands and characteristics of the HSI. For the Lake Erie images and Jasper image dataset, the best number of PCA bands is found to be three. The best ML classifier is found to be the GB classifier for both the Lake Erie and Jasper HSIs. A lower number of PCA bands implies a lesser running time of the models. In the AWS cloud server, the models run in about 6 h and 47 min compared to a regular PC which takes a week for training the models and classification.

## Figures and Tables

**Table 1:** The classification accuracy for HSI2 image 1 using PCA 3, 5, and 7 bands and the different scaling and normalization methods using the four machines for a 1500 pixels batch size.

	Accuracy			
	SVM	LP	GB	GC
PCA-3 ns	68.46	56.88	100.00	87.77
PCA-3 ms	72.67	56.88	100.00	99.94
PCA-3 sc	72.67	56.88	100.00	99.90
PCA-5 ns	63.80	56.88	99.65	81.63
PCA-5 ms	58.55	56.88	99.97	98.87
PCA-5 sc	58.55	56.88	99.93	97.77
PCA-7 ns	58.75	56.88	98.95	79.13
PCA-7 ms	58.07	56.88	99.92	97.26
PCA-7 sc	58.07	56.88	99.97	97.26

**Table 2:** The F1 score for classification of HSI2 image 1 using PCA 3, 5, and 7 bands and the different scaling and normalization methods using the four machines.

	F1-Score			
	SVM	LP	GB	GC
PCA-3 ns	61.80	41.22	100.00	89.75
PCA-3 ms	70.43	41.22	100.00	100.00
PCA-3 sc	70.43	41.22	100.00	99.92
PCA-5 ns	54.97	41.22	99.79	83.41
PCA-5 ms	44.46	41.22	99.90	90.06
PCA-5 sc	44.46	41.22	99.95	85.85
PCA-7 ns	43.64	41.22	99.21	79.53
PCA-7 ms	43.64	41.22	99.95	88.56
PCA-7 sc	43.64	41.22	99.88	87.96

**Table 3:** The classification accuracy for HSI2 image 2 using PCA 3, 5, and 7 bands and the different scaling and normalization methods using the four machines for a 1500 pixels batch size.

	Accuracy			
	SVM	LP	GB	GC
PCA-3 ns	83.97	52.88	100	98.46
PCA-3 ms	53.54	53.54	100	94.94
PCA-3 sc	53.54	53.54	100	93.76
PCA-5 ns	64.81	52.88	99.81	94.74
PCA-5 ms	52.88	52.88	98.19	88.75
PCA-5 sc	52.88	52.88	98.64	88.16
PCA-7 ns	56.58	52.88	86.47	79.98
PCA-7 ms	52.88	52.88	86.88	79.7
PCA-7 sc	52.88	52.88	87.08	79.95

**Table 4:** The F1 score for classification of HSI2 image 2 using PCA 3, 5, and 7 bands and the different scaling and normalization methods using the four machines.

	F1-Score			
	SVM	LP	GB	GC
PCA-3 ns	79.12	36.58	100	98.46
PCA-3 ms	48.25	48.25	100	94.91
PCA-3 sc	48.25	48.25	100	93.78
PCA-5 ns	61.17	36.58	99.81	94.73
PCA-5 ms	36.58	36.58	98.18	88.78
PCA-5 sc	36.58	36.58	98.63	88.19
PCA-7 ns	52.09	36.58	86.22	79.8
PCA-7 ms	36.58	36.58	86.33	80.23
PCA-7 sc	36.58	36.58	86.57	80.53

**Table 5:** The classification accuracy for CyanoHAB HSI using PCA 3, 5, and 7 bands, with the three types of scaling using the four machines for a 1000 pixels batch size.

	Accuracy				
	SVM	LP	GB	GC	
PCA-3 ns	63.48	53.88	99.92	91.53	PCA-3
PCA-3 ms	53.88	53.88	99.33	90.15	
PCA-3 sc	53.71	53.88	98.53	83.73	
PCA-5 ns	61.03	50.04	96.33	76.93	PCA-5
PCA-5 ms	50.04	50.04	97.36	64.18	
PCA-5 sc	49.89	50.04	97.08	74.11	
PCA-7 ns	50.13	48.39	90.71	67.78	PCA-7
PCA-7 ms	48.39	48.39	95.11	56.17	
PCA-7 sc	48.81	48.39	91.25	62.66	

**Table 6:** The F1 score for classification of CyanoHAB HSI using PCA 3, 5, and 7 bands with the three types of scaling using the four machines.

	F1-Score				
	SVM	LP	GB	GC	
PCA-3 ns	53.76	43.52	99.92	91.32	PCA-3
PCA-3 ms	43.52	43.52	99.33	89.50	
PCA-3 sc	43.38	43.52	98.54	83.25	
PCA-5 ns	51.30	37.91	96.35	77.70	PCA-5
PCA-5 ms	37.91	37.91	97.38	64.13	
PCA-5 sc	37.80	37.91	97.10	73.01	
PCA-7 ns	38.49	35.18	90.88	64.37	PCA-7
PCA-7 ms	35.18	35.18	95.14	57.10	
PCA-7 sc	40.19	35.18	91.60	63.39	

**Table 7:** The classification accuracy for Jasper HSI using PCA 3, 5, and 7 bands, with maximum scaling using the four machines for a 300 pixels batch size.

	Accuracy				
	SVM	LP	GB	GC	
Tree	63.60	60.54	95.02	82.76	PCA-3
Water	69.73	55.17	96.93	77.78	
Soil	62.07	66.28	89.27	72.80	
Tree	58.62	58.62	77.47	72.41	PCA-5
Water	61.61	55.17	93.10	69.20	
Soil	64.14	63.45	85.29	71.49	
Tree	58.62	59.44	68.80	68.97	PCA-7
Water	55.83	55.17	76.52	64.20	
Soil	60.26	62.73	80.13	69.13	

**Table 8:** The F1 score for classification of Jasper HSI using PCA 3, 5, and 7 bands with maximum scaling using the four machines.

	F1-Score				
	SVM	LP	GB	GC	
Tree	54.67	47.31	95.03	82.93	PCA-3
Water	64.27	39.23	96.90	78.86	
Soil	51.64	58.98	89.35	71.67	
Tree	43.33	43.33	77.98	69.97	PCA-5
Water	57.04	39.23	93.16	70.95	
Soil	61.99	53.56	86.38	71.84	
Tree	43.33	45.12	69.64	64.98	PCA-7
Water	52.00	39.23	76.87	65.12	
Soil	59.43	51.93	82.53	66.53	

## Data Availability

The Lake Erie hyperspectral image datasets are available at: https://oceandata.sci.gsfc.nasa.gov/directaccess/HSI-HABS-RAW/ (accessed on 14 December 2021). The Jasper hyperspectral image dataset is available at: http://lesun.weebly.com/hyperspectral-data-set.html (accessed on 14 December 2021).
